# A national cohort study on pediatric Behçet’s disease: cross-sectional data from an Italian registry

**DOI:** 10.1186/s12969-017-0213-x

**Published:** 2017-12-21

**Authors:** Romina Gallizzi, Caterina Pidone, Luca Cantarini, Martina Finetti, Marco Cattalini, Giovanni Filocamo, Antonella Insalaco, Donato Rigante, Rita Consolini, Maria Cristina Maggio, Adele Civino, Silvana Martino, Alma Nunzia Olivieri, Giovanna Fabio, Serena Pastore, Angela Mauro, Diana Sutera, Giuseppe Trimarchi, Nicolino Ruperto, Marco Gattorno, Rolando Cimaz

**Affiliations:** 1 Unit of Pediatrics, Department of Human Pathology in Adulthood and Childhood “G. Barresi”, University of Messina, Messina, Italy; 2 Rheumatology Unit Policlinico “Le Scotte”, University of Siena, Siena, Italy; 3 Unit of Pediatrics II, Gaslini Institute, Genoa, Italy; 4 Pediatric Clinic University of Brescia and Spedali Civili of Brescia, Brescia, Italy; 5 Pediatric Rheumatology, Fondazione IRCCS Ca’ Grande, Ospedale Maggiore, Policlinico, Milan, Italy; 6 Department of Pediatric Medicine, Division of Rheumatology, Bambino Gesù Children’s Hospital, Rome, Italy; 7 Institute of Pediatrics, Università Cattolica Sacro Cuore, Fondazione Policlinico Universitario, “A. Gemelli”, Rome, Italy; 8 Unit of Pediatrics, A.O.U, Pisa, Italy; 9 Ospedale dei Bambini “G. Di Cristina, Palermo, Italy; 10 Azienda Ospedaliera Card. G. Panico, Tricase, Lecce, Italy; 11 Unit of Pediatrics, Ospedale Regina Margherita, Torino, Italy; 12 Second University Of Study of Napoli, Naples, Italy; 13 Fondazione IRCCS Ca’ Grande Ospedale Maggiore, Policlinico, Milan, Italy; 14 IRCCS Burlo Garofalo, Trieste, Italy; 15 Università di Messina Dipartimento di Economia Messina, Messina, Italy; 16 Institute “G. Gaslini”, UO Pediatria II, Genoa, Italy; 17 Pediatric Rheumatology Unit, AOU Meyer, University of Florence, Florence, Italy

**Keywords:** Behçet’s disease, Children, Clinical features, Diagnostic criteria, Treatment

## Abstract

**Background:**

Behçet’s disease is a rare multi-systemic inflammatory disease with unknown etiology which involves principally oral and genital mucosa, skin and eyes. Average age at onset of the disease is about 25-30 years, but it may be diagnosed before the age of 16. It is not very rare in Italy, even though there are limited data concerning epidemiology. Aim of this study is to describe the baseline data of an Italian cohort of patients with as having BD or probable BD.

**Methods:**

We described the baseline data of the first national epidemiological study on children coming from 16 Italian Pediatric Rheumatologic Centers diagnosed by the treating physicians as having Behçet’s Disease. Data on demographic characteristics, clinical features and therapy were collected. We then compared our findings to those of international pediatric cohort studies and also retrospectively evaluated the ability to diagnose BD using ISG, ICBD and, for the first time, the new PEDBD criteria.

**Results:**

The study included 110 patients (62 M, 48F). Average age at onset was 8.34±4.11 years. The frequencies of signs/symptoms were: recurrent oral aphtosis 94.5%, genital ulcers 33.6%, ocular 43.6%, gastrointestinal 42.7%, musculoskeletal 42.7%, neurological 30.9% and vascular involvement 10%. Thirty-two patients (29.1%) fulfilled ISG, 78 (70.9%) ICBD, 50 (45.5%) PEDBD criteria and 31 (28%) didn’t fulfill any of them. The most frequently used treatments were colchicine and corticosteroids followed by immunosuppressants. Four patients received biologic therapy (anti TNF-α and anti-IL-1) to treat severe organ involvement.

**Conclusions:**

Recurrent oral aphtosis was the most frequent clinical manifestation, followed by ocular involvement. Gastrointestinal lesions were more frequent in Italy than in non-European countries as opposed to genital ulcers. Skin, ocular and vascular manifestations had a higher frequency in males and genital ulcers in females. Constitutional symptoms were present in 44.5% and recurrent fever in one third of our population.

## Background

Behçet’s disease (BD) is defined as a multi-systemic inflammatory disease with unknown etiology and chronic recurrent pattern, characterized by oral and genital aphthous ulcerations, ocular, skin, articular, vascular, gastrointestinal and central nervous system lesions. BD is included both in vasculitis and autoinflammatory diseases classifications [1]. It is considered a vasculitis affecting vessels of all sizes and defined as a multifactorial autoinflammatory syndrome [2, 3]. Average age at onset of the disease is about 25-30 years, but it may be diagnosed in children before the age of 16 in about 4-26% of cases. Sex distribution is roughly equal, with a worse clinical course in men [4]. BD is prevalent in “Silk Road” populations from East (China) to West (Spain and Portugal): 100/100.000 in China and Iran and 80-370/100.000 in Turkey [5]. Disease occurrence is far lower in European countries such as France, Germany, Sweden, and Italy [6, 7]. Epidemiological studies show a higher prevalence among the southern Italian population compared to the north of Italy: 15.9/100.000 in Potenza (Southern Italy) and 3.80/100.000 in Reggio Emilia (Northern Italy) [6, 8], probably because the south of Italy is part of the “Silk Road”. Etiology is still unknown; pathogenetic mechanisms involve interactions between genetic and environmental factors. The association with human leukocyte antigen (HLA)-B51 is known as the strongest associated genetic risk factor. Furthermore, it has recently been discovered that the haploinsufficiency of A20 protein is related to a “Behçet-like” phenotype with autosomal dominant inheritance [9]. T-lymphocytes play a role, in particular those secreting Interleukin (IL)-17 and IL-21, mostly in acute attacks of BD [7]. There are no pathognomonic tests nor biomarkers to make the diagnosis, which is based on clinical criteria. The most current criteria used are those developed by an international study in 1990 called the International Behçet’s Study Group (ISG) [10] with 85% sensitivity and 96% specificity. Recently, International Criteria for Behçet’s Disease (ICBD) [11] have been validated in adults, with 94.8% sensitivity and 90.5% specificity. Considering these results, the latter should be used as the gold standard. Neither of them has been validated in children. Recently, an international expert consensus group, the Pediatric BD group (PEDBD) has proposed a new set of criteria for the diagnosis of Behçet’s Disease in children. This new international PEDBD criteria has higher sensitivity (91.7%), but lower specificity (42.9%) when compared to ISG [12, 13]. Treatments include topical and systemic corticosteroids, colchicine, immunosuppressants and biological therapy. The therapeutic choice and prognosis depend on clinical involvement. Loss of visual acuity and neurological disease are major causes of morbidity and disability. The primary aim of this study was to collect information on demographic, clinical and therapeutic data from pediatric patients with BD enrolled in the Eurofever registry by Italian Pediatric Rheumatology Centers and to compare these data with other international pediatric studies. A secondary aim was to explore the ability to diagnose BD through the ISG, ICBD and PEDBD criteria in this pediatric cohort. In this study we report the baseline data of a cohort of Italian patients with BD or probable BD enrolled in a longitudinal registry.

## Methods

**Fig. 1:**
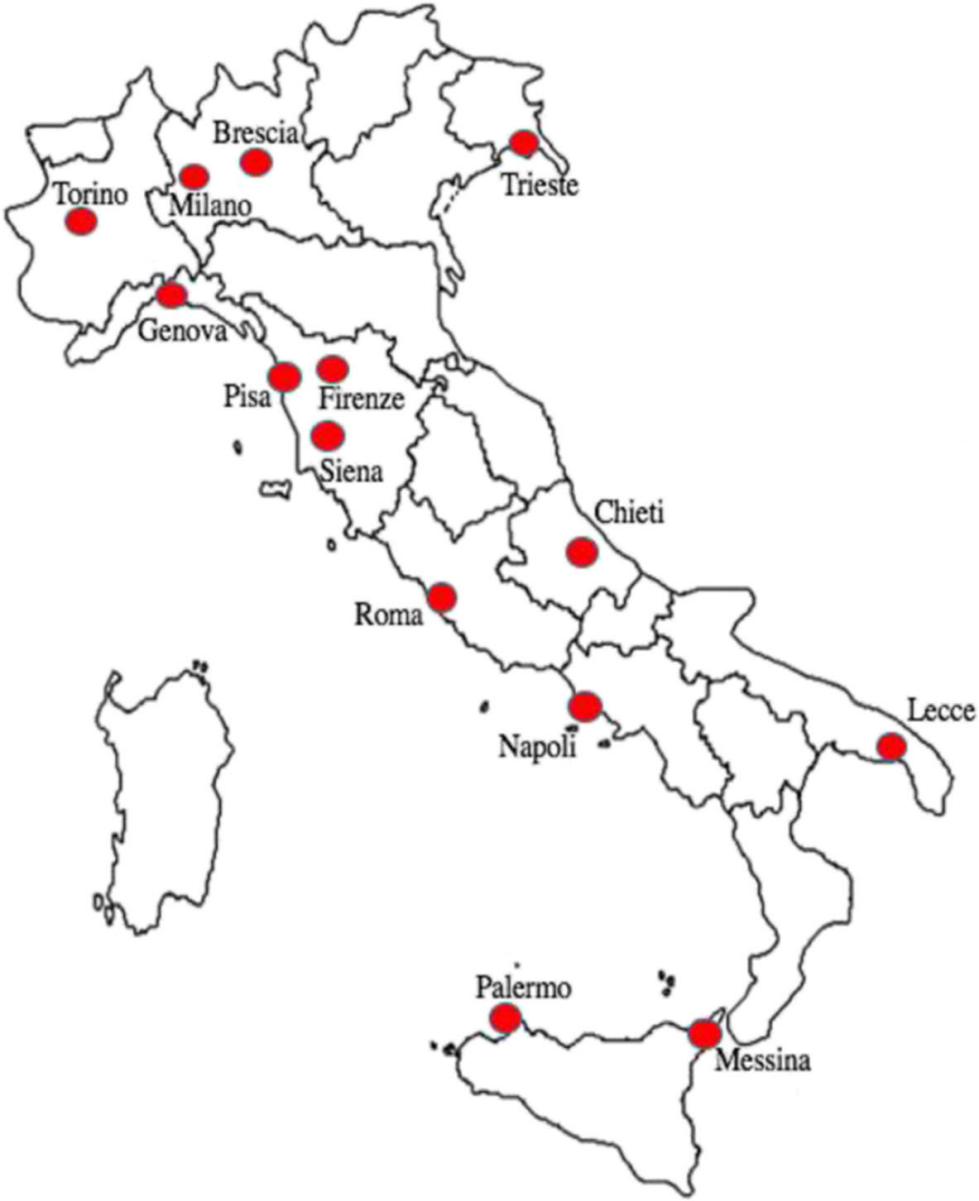
Italian Pediatric Rheumatologic Centers

All patients came from sixteen Italian Pediatric Rheumatologic Centers of fourteen cities (Fig. 1) and the data analyzed in our study were extracted from the Eurofever registry, collected through a secured registry on a https platform hosted in the Paediatric Rheumatology INternational Trials Organization (PRINTO) Website (https://www.printo.it/). Ethical committee approval for entering patients in the registry and informed consent were obtained in the participating centers as per local regulations. We included consecutive pediatric patients affected by BD who met ISG or were diagnosed by physician as being affected by BD or probable BD. Disease onset after the age of 16 was considered a reason for exclusion. Patients were diagnosed and entered in the registry by their local physician. In case of inconsistency or other doubts, specific queries were resubmitted to the participating centers for resolution. Data extracted included the following information: demographics (sex, age at onset, age at diagnosis), ethnicity, family history, clinical manifestations (mucocutaneous, musculoskeletal, ocular, gastrointestinal, vascular, neurological manifestations), the presence of HLA B51 and treatment. We tested the applicability of ISG, ICBD and PEDBD criteria in our cohort.

### Statistical analysis

All quantitative and qualitative variables are reported as mean and standard deviation. Assumption of normal distribution for continuous variables was tested by Shapiro-Wilk test. Non-normally distributed variables were compared by Mann-Whitney U test. The Chi-square test was utilized to assess the association between clinical manifestations (mucocutaneous, ocular, gastrointestinal, vascular, neurological involvement) and gender and also HLA B51. A *p* value <0.05 was considered statistically significant. All the processing was done using the R software (https://www.r-project.org/). The frequency of patients who met ISG, ICBD and PEDBD criteria was evaluated.

## Results

One hundred and ten patients were included in our study, 62 males and 48 females, coming from 16 Italian Pediatric Rheumatologic Centers of 14 cities. There were familial cases in 12% of our patients. Average age at onset was 8.34 ± 4.11 years and average age at diagnosis 11.29 ± 3.95 years. All patients were Caucasians.

## Clinical features

**Fig. 2:**
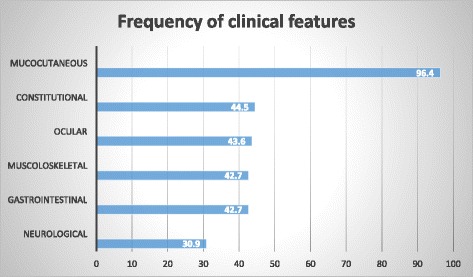
Frequency of clinical features

Mucocutaneous symptoms were present in 106/110 patients (96.4%), isolated skin lesions in 36 (39.6%); ocular involvement in 48 (43.6%), gastrointestinal (GI) in 47 (42.7%); musculoskeletal in 47 (42.7%); neurological in 34 (30.9%), vascular in 2 (1.8%) and constitutional symptoms in 49 (44.5%). Among mucocutaneous manifestations, the most common was recurrent oral aphtosis (ROA) in 104 (94.5%) followed by genital ulcers (GU) in 37 (33.6%), pseudo-follicolitis in 16 (14.5), erythema nodosum in 15 (13.6%), papulo-pustular lesions in 13 (11.8%) and acneic lesions in 12 (10.9%). Pathergy test was positive in 14.5% of cases in which it was performed (15/103). Ocular involvement occurred in 48 patients: 27 (24.5%) as anterior uveitis, 14 (12.7%) posterior uveitis, 9 (8.2%) retinal vasculitis, 7 (6.4%) papilledema, 5 (4.5%) papillitis, 4 (3.6%) episcleritis and 2 keratitis (1.8%). Arthralgia was present in 45 cases (41%) and arthritis in 25 (22.7%): (14 oligoarthritis, 8 polyarthritis and 3 monoarthritis). Abdominal pain in 45 (41%) and diarrhea in 15 (13.6%) were the most common GI symptoms, 7 (6.4%) had GI bleeding, 5 (4.5%) GI ulcers, 5 (4.5%) anal ulcers and 2 (1.8%) gut perforation. Headache was the main neurologic symptom, being present in 27 cases (24.5%), while 5 (4.5%) showed cranial nerve palsy, 2 (1.8%) cranial neuropathy, 2 (1.8%) aseptic meningitis, 2 (1.8%) optic neuritis, 1 (0.9%) peripheral neuropathy. Venous thrombosis occurred in 2 patients, with thrombosis of transverse sinus in one of them. Constitutional symptoms included recurrent fever in 34 (30.9%), malaise in 32 (29.1%), fatigue in 31 (28.1%) and mood disorder in 7 (6.4%) patients (Fig. 2). Twenty-nine (26.3%) patients had a continuous disease course, 67 (60.9%) recurrent and 14 (12.72%) continuous-recurrent.

## Laboratory findings

HLA-B51 testing was performed in 88 patients and was present in 50 (56.8%). More than half of our patients (54.5%) had increased acute phase reactants (erythrocyte sedimentation rate and C-reactive Protein). Genetic testing for autoinflammatory conditions was performed in 16/110 patients (16/34 with recurrent fever): Familial Mediterranean Fever (FMF) 16/16; Tumor Necrosis Factor Receptor Associated Periodic Syndrome (TRAPS) 13/16 and Mevalonate Kinase Deficiency (MVK) 5/16. Results were negative in all cases.

## Treatment

All patients received topical steroid therapy (ocular and/or mucocutaneous). The most used systemic treatments were colchicine in 33 patients (30%), as monotherapy in 22 and in combination in 11 and corticosteroids in 33 (30%) patients (as monotherapy in 14); immunosuppressants in 13 (11.8%) with the following drugs: 7 Methotrexate, 6 Azathioprine, 6 Cyclosporine, 2 Thalidomide, 1 Cyclophosphamide, 1 Sulphasalazine. Anti-tumor necrosis factor-alpha (anti TNF-α) was used in 2 patients (Infliximab) and anti-IL-1 (Anakinra) in 2. Non Steroidal Anti-Inflammatory Drugs (NSAIDs) were used in 4 cases.

## Diagnostic criteria

Out of the 110 patients, 32 (29.1%) met the ISG, 78 (70.9%) ICBD and 50 (45.5%) PEDBD criteria; 28 (25.5%) met all three, 25 (22.7%) two criteria and 31 (28%) did not meet any of them.

## Discussion

We described the baseline data of the first national epidemiological study on BD in Italian children and compared our findings to those of other international pediatric cohort studies (Table 1). In the literature, there are few epidemiological data on pediatric BD, mostly limited to case reports or case series. In our patients the average age of disease onset was 8.3 ± 4.1 years, similar to most of the studies we analyzed. This age was however earlier in the English population (4.8 years) [14] and later in life in Tunisia (16.1 years) [15], while the average age of diagnosis was 11.2 ± 3.9 years. To date, the main problem remains diagnosis, as BD is characterized by remittance and exacerbations and the symptoms are separated from one another by years, which can result in delay of diagnosis up to 13.5 years [14]. In our study, we noted a diagnostic delay of only 3 years (2.9 ± 3.6), probably since our patients came from pediatric rheumatologic centers of high expertise. In children, even though the first symptoms may start very early, the disease is rarely complete before the age of 16 [15] and, because of a lack of awareness of BD, other better known pathologies are more often hypothesized (e.g. immunodeficiency, gastrointestinal diseases).

**Table 1:** Comparison of clinical features of BD in different geographical regions

	present study	Davatchi 2010 Iran [20]	Atmaca 2011 Turkey [18]	Karincaoglu 2008 Turkey [21]	Konet-Paut 1998 International study [17]	Sungur 2009 Turkey [14]	Hamzaoui 2014 Tunisia [13]	Kim 1994 Korea [19]	Brogan 2016 UK [12]
Total number of PEDBD patients	110	1973	110	83	86	62	81	40	46
Male/female ratio	1.3	1.0	0.6	0.8	1.0	1.1	2.1:1	0.67	0.9
Average age at onset	8.3	n.r.	11.6 ± 3.4	12.3	8.4	n.r.	16.12 ± 3.7	n.r.	4.87
ROA	94.5^a^	97.8	100	100	97.2	100	100	100	97.8
GU	33.6	64.7	82.7	82	60.4	55	76.5	82.5	74
Skin manifestations	39.6	65.3	76	n.r.	93	n.r.	88.9	72.5	32.6
Ocular involvement	43.6	56.1	30.9	35	57	n.r.	44.4	27.5	8.7
Vascular manifestations	1.8	6.5	3.6	9.6	16.2	5	32.1	n.r.	6.5
Joint involvement	42.7	37.1	22.7	40	45.3	42	40.7	27.5	52
CNS	30.9	10.3	3.6	7.2	36	13	22.2	2.5	32.6
GI lesions	42.7	7.6	n.r.	4.8	14	n.r.	n.r.	5	58.7
Pathergy phenomenon	14.5^1^	49.4	45.5	37	63.2^2^	47	55.7	17.5	60^3^
Relatives affected	12^4^	n.r.	12.3	19	16.2	42	n.r.	22.5	17

*ROA* Recurrent oral aphtosis, *GU* Genital ulcers, *CNS* central nervous system, *GI* Gastrointestinal, *nr* not reported

^a^all clinical manifestations are expressed in percentage

^1^Pathergy test was performed in 103/110 patients and was positive in 15/103

^2^Pathergy test was performed in 68/86 patients and was positive in 43/86

^3^Pathergy test was performed in 5 patients and was positive in 3/5

^4^Data not known in 10 patients; 12/110 patients had a positive family history

The sex ratio was not consistent among the studies analyzed. Familial cases can occur, with figures ranging widely from 12% in our study to 42% in Turkey [16]. According to Koné-Paut et al. the frequency of familial cases is significantly higher (*p* < 0.0001) in pediatric BD patients (12.3%) than in non-pediatric patients (2.2%) and this suggests a strong genetic component [17]. A genetic influence on disease predisposition is also supported by the recent discovery of a familial form (A20 gene), which has the clinical manifestations of BD in multiple family members.

ROA is by far the most frequent clinical manifestation, with a lower rate in our population than in all the other studies. Ocular involvement is the second manifestation by frequency in our cohort (43.6%), while it has a high variability in the other populations, from 8.7% in UK to 81% in Turkey [16], even if the latter finding come from a study performed in an Ophthalmology Department. Skin involvement rate in our patients was similar to those from UK and much lower than those from all other countries. Vascular manifestations were seen with the highest frequency in Tunisia (32.1%) [15], much higher when compared to our series (1.8%). Vascular involvement has a wide range (5-40%) in the literature and this could be due to the difference between reference centers, patients with different disease duration and ethnic variations, being quite rare in the Far-East [18]. In our cohort, the percentage of neurological involvement was 30.9% similar to the study by Kone-Paut [19] and Brogan [14]; other studies had a significantly lower involvement rate. These differences may be due to the fact that in countries with a higher percentage of neurological involvement, isolated headaches were considered a neurologic symptom, although it is not included among the criteria for the diagnosis of Neuro-Behçet. In our study, isolated headaches were present in 24.5% of cases. In the PEDBD study [12], the presence of headaches was significantly associated with BD confirmation by experts (*p* = 0.0063). Joint involvement was reported with a percentage of about 40% in all studies evaluated, including ours, but was lower in a Turkish study [20] and in one from Korea [21]. GI involvement was frequent in our series and in UK cohort with a rate of 42-58%, much lower in all other studies (4.8-14%) [19, 21–23]. Arthralgia (24.5%) and abdominal pain (22.7%) without any organic involvement were very frequent in our population. Pathergy phenomenon was known to be present mostly in Turkey, Tunisia and Iran [15, 16, 22] (49.4-63.2%); in our Caucasian series it was 14.5%. This data cannot be compared to the other Caucasian population in UK, since it was performed in only 5 out of 46 patients. We also assessed constitutional symptoms and recorded a rate of 28% of fatigue and malaise, while 6.4% patients complained of mood disorders. Thirty-four patients had recurrent fever with irregular pattern.

We have assessed the correlations between clinical findings and gender without finding statistically significant data, even if we have found a greater frequency of skin, ocular and vascular manifestations in males and GU in females. We also assessed all the clinical findings and disease onset age and discovered that patients with neurological involvement had a higher average age of BD onset than those who did not (*p* = 0.002). We also recorded similar data for vascular involvement, though not statistically significant (*p* = 0.071). This kind of correlation has not been assessed in the other studies. The presence of HLA B51 was (56.8%) similar to Tunisia and its presence was higher in patients with ocular involvement (*p* = 0.219). Our results overall are very similar to the UK study and differ from other studies coming from Silk Road, highlighting a key role of racial differences and/or environmental factors.

We employed ISG, ICBD and, for the first time, PEDBD criteria with our patients and demonstrated a sensitivity of 29.1% with ISG, 70.9% ICBD and 45.5% PEDBD. These data confirm the limits of ISG, the greater sensitivity of ICBD and PEDBD, albeit lower in the latter case. A limit of ISG is ROA as a mandatory criterion and the absence of neurological and vascular involvement; on the contrary, ICBD have no mandatory criteria and neurological and vascular manifestations have been added, as well as in PEDBD. The last two criteria give a different weight to every item: 2 points for ROA, GU and ocular lesions and 1 point for all the others in ICDB and 1 point for each one in PEDBD. This means that only two items are needed to diagnose BD with ICBD, but 3 items with PEDBD. Among our 110 patients, 31 (28%) were defined by the experienced centers as suspected or probable BD even if they did not fulfill any of the three criteria used: 18 had only ROA, 7 ROA and skin lesions, 5 only ocular manifestations and 1 had only skin lesions. In most of them, the positive family history, the presence of a chronic or recurrent systemic inflammation, the presence of HLA-B51 and the response to treatment (mainly colchicine) was the main reason to include them in the registry. Notably, in most of these patients the administration of a continuous treatment clearly influenced the natural clinical course of the disease. It is therefore difficult to clearly determine if these patients would have the chance to develop other BD-related clinical manifestations in their follow-up. A longitudinal study, already planned, will provide evidence of the proportion of these patients that will reach a definitive diagnosis. In any case, our cohort identifies the existence of a sort of “gray-zone” of pediatric patients that do not fulfill any of the clinical criteria for BD, but must be treated with the same therapeutic approaches used in patients that fulfill at least one of the ongoing diagnostic/classification criteria.

BD enters in differential diagnosis with autoinflammatory diseases (AID), so we assessed constitutional symptoms in our cohort and recorded recurrent fever in one third of our population (34 patients): 16 of them have also been studied for the most common AID (FMF, TRAPS, HIDS) without finding any mutations. In a PEDBD cohort of Kone-Paut, 44% of children had recurrent fever, a sign mostly associated to vascular and neurological disease, but also observed in association with attacks of ROA, which may seem to be Periodic Fever Aphtosis Pharyngitis and Adenitis (PFAPA) syndrome [9].

In BD, there is no approved treatment guideline and a specific therapy based on organ involvement on a case-by-case basis is usually carried out. All our patients received topical steroid therapy (ocular and/or mucocutaneous). The systemic treatments more commonly used were colchicine and corticosteroids, followed by immunosuppressants. All patients who took colchicine as monotherapy, presented a clinical picture characterized by ROA, frequently associated with GU and skin lesions. Patients who received immunosuppressive therapy with or without colchicine had all severe ROA, to a lesser degree GU, but many of them had ocular involvement. Four patients received biologic therapy: 2 of them anti TNF-α (Infliximab) and 2 anti IL-1 (Anakinra), for severe ocular, neurological, GI and mucocutaneous involvement. Among the populations we evaluated, only UK study used anti TNF-α agents. Some pediatric case reports and case series described the efficacy and safety of TNF-α inhibitors to manage many BD manifestations; more recently IL-1 blockade have been successfully used to treat severe BD cases and/or refractory to anti TNF-α [24–27]. Many of our patients received drug combinations and twenty of them needed to change two, three or even four medication types.

## Conclusion

We described the baseline data of the first national epidemiological study on pediatric BD in Italy. We noted a high frequency of familial cases, confirming a strong genetic influence. ROA is by far the most frequent clinical manifestation, followed by ocular involvement; GI lesions are more frequent in Italy than in non-European countries as opposed to GU. We also recorded a higher frequency of skin, ocular and vascular manifestations in males and GU in females. Constitutional symptoms, rarely mentioned before, worsen definitely quality of life. Recurrent fever was present in one third of our population. Our data confirm a better sensitivity of ICBD and PEDBD in children when compared to ISG criteria. Colchicine and corticosteroids were the main treatments and some severe cases received biologic agents (anti TNF-α and anti-IL-1). The study suffers for the lack of longitudinal data. Thus the results refer only on the clinical manifestations and treatments from disease onset to the enrollment in the national cohort. Longitudinal data will provide precious information on the evolution of the disease and on the efficacy and safety of different treatments used in this condition.

## Abbreviations

AID: Autoinflammatory diseases
anti TNF-α: Anti Tumor Necrosis Factor – alpha
BD: Behçet’s disease
CNS: Central nervous system
FMF: Familial mediterranean fever
GI: Gastrointestinal
GU: Genital ulcers
HLA: Human leukocyte antigen
ICBD: International Criteria for Behcet’s Disease
IL: Interleukin
ISG: International Behcet’s Study Group
MVK: Mevalonate kinase deficiency
NSAIDs: Non steroidal anti-inflammatory drug
PEDBD: Pediatric BD
PFAPA syndrome: Periodic fever aphtosis pharyngitis and adenitis syndrome
PRINTO: Paediatric rheumatology international trials organisation
ROA: Recurrent oral aphtosis
TRAPS: Tumor necrosis factor receptor associated periodic syndrome
UK: United Kingdom
