# Correction to: A national cohort study on pediatric Behçet’s disease: cross-sectional data from an Italian registry

**DOI:** 10.1186/s12969-018-0241-1

**Published:** 2018-04-23

**Authors:** Romina Gallizzi, Caterina Pidone, Luca Cantarini, Martina Finetti, Marco Cattalini, Giovanni Filocamo, Antonella Insalaco, Donato Rigante, Rita Consolini, Maria Cristina Maggio, Adele Civino, Silvana Martino, Alma Nunzia Olivieri, Giovanna Fabio, Serena Pastore, Angela Mauro, Diana Sutera, Giuseppe Trimarchi, Nicolino Ruperto, Marco Gattorno, Rolando Cimaz, for EUROFEVER and the Paediatric Rheumatology International Trials Organisation (PRINTO)

**Affiliations:** 1 Unit of Pediatrics, Department of Human Pathology in Adulthood and Childhood “G. Barresi”, University of Messina, Messina, Italy; 2 Rheumatology Unit Policlinico “Le Scotte”, University of Siena, Siena, Italy; 3 Unit of Pediatrics II, Gaslini Institute, Genoa, Italy; 4 Pediatric Clinic University of Brescia and Spedali Civili of Brescia, Brescia, Italy; 5 Pediatric Rheumatology, Fondazione IRCCS Ca’ Grande, Ospedale Maggiore, Policlinico, Milan, Italy; 6 Department of Pediatric Medicine, Division of Rheumatology, Bambino Gesù Children’s Hospital, Rome, Italy; 7 Institute of Pediatrics, Università Cattolica Sacro Cuore, Fondazione Policlinico Universitario, “A. Gemelli”, Rome, Italy; 8 Unit of Pediatrics, A.O.U, Pisa, Italy; 9 Ospedale dei Bambini “G. Di Cristina, Palermo, Italy; 10 Azienda Ospedaliera Card. G. Panico, Tricase, Lecce Italy; 11 Unit of Pediatrics, Ospedale Regina Margherita, Torino, Italy; 12 Second University Of Study of Napoli, Naples, Italy; 13 Fondazione IRCCS Ca’ Grande Ospedale Maggiore, Policlinico, Milan, Italy; 14 IRCCS Burlo Garofalo, Trieste, Italy; 15 Università di Messina Dipartimento di Economia Messina, Messina, Italy; 16 Institute “G. Gaslini”, UO Pediatria II, Genoa, Italy; 17 Pediatric Rheumatology Unit, AOU Meyer, University of Florence, Florence, Italy

## Correction

Following publication of the original article [1], the authors reported that the names of two institutional authors – EUROFEVER and the Paediatric Rheumatology International Trials Organisation (PRINTO) – had been unintentionally omitted in the final online version of the manuscript. The corrected author list is shown in this Correction.
